# Cognitive performance in patients with ischemic stroke and additional myocardial injury – results from the multicenter prospective observational PRAISE study

**DOI:** 10.1186/s42466-025-00446-4

**Published:** 2025-11-04

**Authors:** Regina von Rennenberg, Simon Litmeier, Kristina Szabo, Annerose Mengel, Martina Petersen, Silke Wunderlich, Dominik Michalski, Götz Thomalla, Bernd Kallmünzer, Gabor Petzold, Martin Dichgans, Timo Siepmann, Georg Royl, Peter Arthur Ringleb, Christian H. Nolte, Matthias Endres

**Affiliations:** 1 Department of Neurology with Experimental Neurology, Charité-Universitätsmedizin Berlin, Campus Benjamin Franklin, Hindenburgdamm 30, 12203 Berlin, Germany; 2 Center for Stroke Research Berlin (CSB), Berlin, Germany; 3 German Center for Neurodegenerative Diseases (DZNE) Partner Site, Berlin, Germany; 4 Department of Neurology, Mannheim Center for Translational Neuroscience, Medical Faculty Mannheim, Department of Neurology, Mannheim, Germany; 5 Department of Neurology and Stroke, University Tübingen, Tübingen, Germany; 6 Department of Neurology, Klinikum Osnabrück, Osnabrück, Germany; 7 Department of Neurology, School of Medicine and Health, Technical University of Munich, Klinikum Rechts der Isar, Munich, Germany; 8 Department of Neurology, Leipzig University, Leipzig, Germany; 9 Klinik und Poliklinik für Neurologie, Kopf- und Neurozentrum, Universitätsklinikum Hamburg-Eppendorf, Hamburg, Germany; 10 Department of Neurology, Universitätsklinikum Erlangen, Friedrich-Alexander- Universität Erlangen-Nürnberg, Erlangen, Germany; 11 Department for Vascular Neurology, Universitätsklinikum Bonn, Bonn, Germany; 12 German Center for Neurodegenerative Diseases (DZNE), Bonn, Germany; 13 Institute for Stroke and Dementia Research (ISD), University Hospital, LMU Munich, Munich, Germany; 14 Department of Neurology, Medical Faculty, University Hospital Carl Gustav Carus, TUD Dresden University of Technology, Dresden, Germany; 15 Department of Neurology, University Medical Center Schleswig-Holstein, Campus Lübeck, Germany; 16 Department of Neurology, University Hospital Heidelberg, Heidelberg, Germany; 17 German Centre for Cardiovascular Research (DZHK) Partner Site, Berlin, Germany; 18 Berlin Institute of Health (BiH), Berlin, Germany; 19 German Center for Mental Health (DZPG), Partner Site, Berlin, Germany

## Abstract

**Background:**

In the general population, cognitive impairment and dementia are more common in individuals with prior myocardial injury, defined as elevated levels of high-sensitive cardiac troponin (hs-cTn). In stroke patients, data on the link between myocardial injury and cognitive outcome are scarce. We aimed to analyze the association between the severity of myocardial injury (degree of hs-cTn elevation), presence of acute myocardial injury (dynamic change in elevated hs-cTn values > 20% in serial measurements) and cognitive performance over time after acute ischemic stroke.

**Methods:**

This is a prespecified analysis of the prospective multicenter observational PRediction of Acute coronary syndrome in acute Ischemic StrokE (PRAISE) study. PRAISE included 254 patients with an acute ischemic stroke or transient ischemic attack (TIA) and myocardial injury in 26 centers in Germany. Patients underwent cognitive assessment at baseline and before hospital discharge using the Montreal Cognitive Assessment (MoCA) and at three and twelve months after the index event using the Telephone Interview for Cognitive Status (TICS). We used linear regression to analyze the associations between cognitive performance and (1) severity of myocardial injury and (2) presence of acute myocardial injury. The association between hs-cTn and TICS scores over time was examined using inverse probability weighted generalized linear models.

**Results:**

Severity of myocardial Injury was associated with lower MoCA scores (adjusted beta − 2.6, 95% CI -4.0 - -1.2, *p* < 0.001) and higher proportion of cognitive impairment (i.e. MoCA score < 26 points) (adjusted OR 2.9, 95%CI 1.3–6.7, *p* = 0.012). Acute myocardial injury was associated with better cognitive performance (adjusted beta 1.8, 95% CI 0.4–3.1, *p* = 0.011). We found no association between hs-cTn and cognitive decline over twelve months.

**Conclusions:**

In patients with ischemic stroke, the severity of myocardial injury in general but not the presence of acute myocardial injury at time of stroke is associated with cognitive impairment.

**Trial registration:**

Clinicaltrials.gov NCT03609385 https://clinicaltrials.gov/study/NCT03609385?term=NCT03609385&rank=1 Date of registration 6th July 2018.

**Supplementary Information:**

The online version contains supplementary material available at 10.1186/s42466-025-00446-4.

## Background

Patients with ischemic stroke are at high risk for cognitive impairment and dementia [1]. Patients with cardiac comorbidites [2] also show cognitive impairment more often. In the general population, even subclinical myocardial injury (as indicated by elevation of high-sensitivity cardiac troponin (hs-cTn) without other clinical evidence of cardiac damage) is linked to cognitive impairment [3, 4].

Although hs-cTn elevation (i.e. myocardial injury) is common in patients with acute ischemic stroke, data on the association of myocardial injury and cognitive performance after stroke are scarce [5, 6]. One study found that higher levels of hs-cTn were associated with poorer cognitive performance over a period of three years but not with steeper cognitive decline in patients with first-ever acute ischemic stroke [7]. Another study found an association between hs-cTn and cognitive decline in domains that are typically affected by vascular cognitive impairment [6]. However, both studies were limited by only a single hs-cTn measurement per patient [6, 7]. Both chronic and acute myocardial injury may occur [8] in acute ischemic stroke patients. Acute myocardial injury is defined by elevated hs-cTn levels with a dynamic change of >20% in serial measurements, while stable levels with elevation above the upper reference limit (URL) indicate chronic myocardial injury [9]. In previous studies, acute myocardial injury in particular has been associated with poor post-stroke outcome, especially higher post-stroke mortality [8, 10]. The differential impact of acute vs. chronic myocardial injury on cognitive outcome after stroke remains unclear. Here, we report the data on both severity and acuteness of myocardial injury (hs-cTn) and cognitive outcome over 12 months in the multicenter PRediction of Acute coronary syndrome in acute Ischemic StrokE (PRAISE) study [11].

## Methods

### Study design

The PRAISE study was a prospective, multicenter, observational study including patients with acute ischemic stroke or high-risk transient ischemic attack (TIA) and elevation of hs-cTn [11, 12]. The protocol of the PRAISE study has been published previously [12]. Patients were included at 26 stroke centers in Germany. Patients were eligible if they were admitted to hospital within 72 h after symptom onset. Diagnosis of acute ischemic stroke was established by clinical examination and cerebral imaging (CT or MRI). Patients who had undergone acute reperfusion therapy for their stroke (i.e. intravenous thrombolysis or mechanical thrombectomy) were required to undergo follow-up imaging prior to inclusion to exclude intracranial hemorrhage. High risk TIA was defined as transient focal neurological deficits verified by a neurologist, absence of an ischemic lesion on cerebral imaging and an ABCD2 score of ≥ 4. Patients with severe renal failure, defined as a glomerular filtration rate < 30 ml/min were not eligible for inclusion. The primary aim was to identify diagnostic criteria for myocardial infarction in acute ischemic stroke patients. Cognitive outcome was a pre-specified secondary endpoint. Patients underwent two study visits during the in-hospital stay. The first study visit (V1) was scheduled directly after study inclusion, within 72 h after hospital admission. The second visit (V2) had to be performed within seven days after inclusion into the study, before hospital discharge. Stroke severity at V1 was measured using the National Institutes of Health Stroke Scale (NIHSS) [13]. Follow-up was scheduled after three and twelve months in form of telephone interviews. Recruitment for the PRAISE study took place between August 2018 and October 2020 and follow-up interviews were completed by December of 2021.

### Laboratory data

**Fig. 1:**
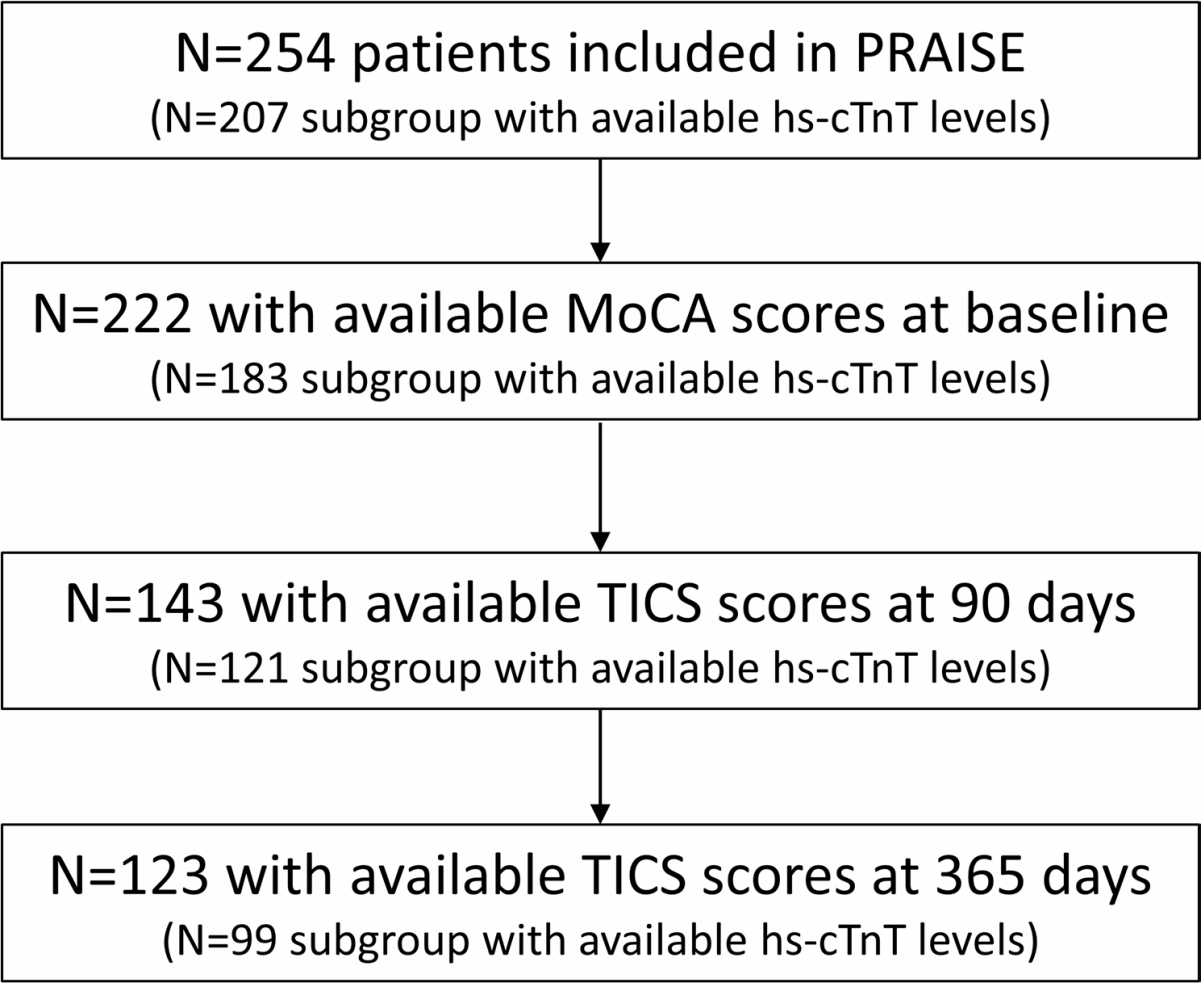
Flowchart for inclusion/exclusion of patients. Abbreviations: hs-cTnT = high-sensitivity cardiac troponin T, MoCA = Montreal Cognitive Assessment, TICS = Telephone Interview for Cognitive Status

Patients were eligible if they fulfilled the European Society of Cardiology (ESC) 0 h/3 h criteria for suspected non-ST elevation myocardial infarction, i.e. either (1) very high initial hs-cTn values (defined as >52 ng/L if hs-cTnT, Roche Elecsys assay, or >52 ng/L, if hs-cTnI, Abbott Architect assay, or >107 ng/L, if hs-cTnI, Dimension Vista assay) or (2) a dynamic change of hs-cTn >20% in repeated measurements with at least one value above the assay-specific URL [14]. Thus, all patients included in the PRAISE study had elevated levels of hs-cTn. Hs-cTn was measured on hospital admission and again after three hours as part of clinical routine in consecutive ischemic stroke patients treated at the participating sites. Study centers used different hs-cTn assays. Therefore, the blood samples were re-analyzed in a central core laboratory using the high-sensitivity Troponin T Roche Elecsys Assay^®^. Thus, hs-cTnT levels were available in a sub-sample of *N* = 207/254 patients (81.5%, see Fig. 1) [11]. This test has a cut-off at 14 ng/l as its URL.

### Cognitive endpoints

Cognitive performance at baseline (V1) and before discharge (V2) was measured using the Montreal Cognitive Assessment (MoCA) [15]. The total MoCA score is a sum of seven subscores given for the different cognitive domains assessed (see supplemental table S1). The MoCA is one of the recommended screening tools for post-stroke cognitive impairment and the validated cut-off (< 26 points) indicates cognitive impairment in stroke patients with high sensitivity and specificity [16]. Cognitive performance at follow-up (i.e. three and twelve months after stroke) was measured using the Telephone Interview for Cognitive Status (TICS) [17]. The TICS is a global cognitive screening tool developed from the Mini Mental Status Examination for telephone administration and ranges from 0 to 41 points [18]. The TICS has been validated in the detection of post-stroke cognitive impairment with similar discriminative ability to the MoCA and T-MoCA [17, 18].

### Statistical analysis

Data are shown as median with interquartile range (25th and 75th percentile) for continuous and absolute (N) and relative (%) frequencies for categorical variables. Since there were missing observations in the TICS score, we compared baseline characteristics of patients with at least one available TICS score to those of patients with no available TICS score using the Mann-Whitney-U test for continuous variables and the Chi-Square test for dichotomous variables.

To analyze the association between hs-cTn levels and cognition we used (1) log-transformed absolute values of hs-cTnT on admission to analyze severity of myocardial injury and (2) presence of acute myocardial injury. We performed unadjusted and adjusted linear regression analyses to examine the association with total MoCA scores and ordinal regression models to estimate the association with the respective constituent MoCA subscores (executive function, naming, attention, language, abstraction, delayed recall and orientation).

To analyze the associations between hs-cTn and TICS scores over time, we calculated generalized linear models with time-specific weights (IPW GLM) using the xtrccipw command in Stata [19]. We used this method since there was a relevant number of missing observations for the TICS measurements (see Fig. 1). Those missing data were handled in the model with time-specific weights on available observations. The weights were set on continuation in the study period. We included time in months on a continuous scale as a time variable and the patient identifier to indicate dependencies of the outcomes due to repeated TICS measurements within one subject. We adjusted all models for age, sex, history of hypertension, history of diabetes, history of coronary artery disease, history of heart failure, history of atrial fibrillation, glomerular filtration rate and baseline NIHSS. Longitudinal analyses also adjusted for cognitive impairment at baseline.

## Results

### Baseline characteristics and clinical outcome

In total, *N* = 254 patients were included in the PRAISE study. Table 1 displays the baseline characteristics of the PRAISE study population stratified by patients with at least one available TICS score vs. patients missing all TICS scores. Overall, 167/222 (75%) patients with available baseline MoCA scores had cognitive impairment at baseline.

**Table 1:** Baseline characteristics and clinical outcome stratified by patients with at least one TICS score and patients missing all TICS scores

	At least one TICS (*n* = 151)	Missing TICS completely (*n* = 103)	*p*
Median age at baseline (IQR)	74 (64–80)	77 (69–83)	0.008*
Female sex, n (%)	69 (45.7%)	51 (49.5%)	0.549
History of hypertension, n (%)	116 (76.8%)	82 (79.6%)	0.598
History of diabetes, n (%)	40 (26.5%)	32 (31.1%)	0.427
History of coronary artery disease, n (%)	40 (26.5%)	29 (28.2%)	0.770
History of heart failure, n (%)	16 (10.6%)	12 (11.7%)	0.771
History of atrial fibrillation, n (%)	33 (21.9%)	37 (35.9%)	0.012*
Median NIHSS (IQR)	1 (0–3)	3 (1–6)	< 0.001*
Median MoCA at baseline (IQR)	23 (20–26) *N* = 137	20 (15–24) *N* = 85	< 0.001
Cognitive impairment at baseline (MoCA < 26), n (%)	92 (60.9%) *N* = 149	75 (72.8%) *N* = 85	< 0.001*
Median MoCA at discharge (IQR)	24 (19–27) *N* = 130	19 (15–23) *N* = 74	< 0.001*
Median initial hs-cTnT (IQR) (*N* = 207)	73 (41–150)	74 (37–173)	0.761
Dynamic change of hs-cTn > 20%, n (%)	85 (56.3%)	55 (53.4%)	0.624
Mortality at twelve months, n (%)	10 (6.6%)	32 (31.1%)	< 0.001
MACE at twelve months, n (%)	21 (13.9%)	42 (40.8%)	< 0.001
Median mRS at twelve months (IQR) (*N* = 218)	1 (0–2) *N* = 146/151	4 (3–6) *N* = 72/103	< 0.001

Univariable comparisons were performed using Chi-Square test for dichotomous variables and Mann-Whitney-U test for continuous variables. Abbreviations: TICS = telephone interview for cognitive status, NIHSS = National institutes of health stroke Scale, IQR = interquartile range, MoCA = Montreal cognitive Assessment, hs-cTnT = high-sensitivity cardiac troponin T, URL = upper reference limit, MACE = major adverse cardiovascular events (i.e. death, recurrent stroke, recurrent myocardial infarction), mRS = modified Rankin Scale. *p-value < 0.05

### Hs-cTn and cognitive performance at baseline and before hospital discharge

**Fig. 2:**
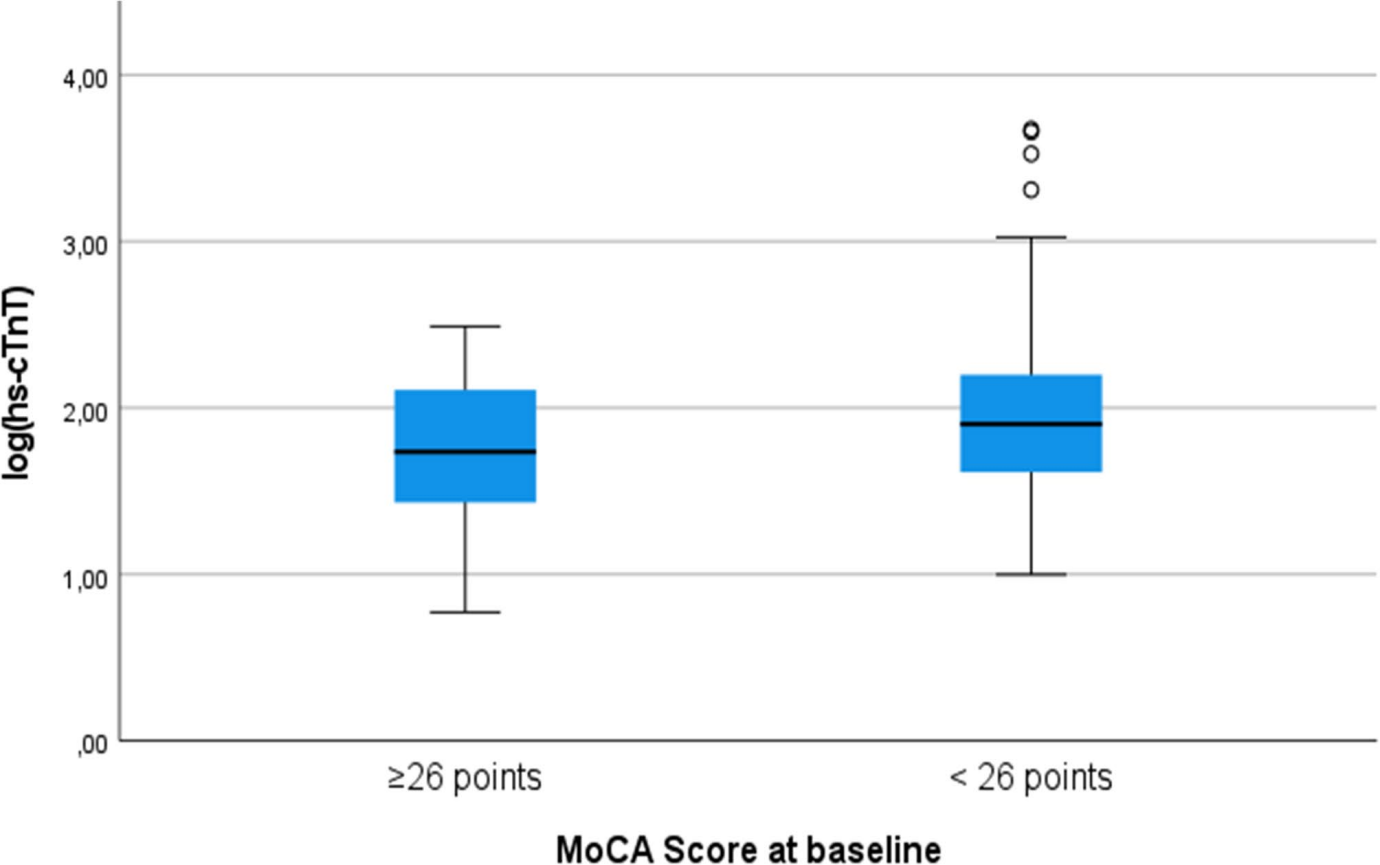
Median hs-cTnT values and interquartile ranges according to presence of cognitive impairment (defined as a MoCA total score < 26 points) at baseline. Abbreviations: MoCA = Montreal Cognitive Assessment, hs-cTnT = high-sensitivity cardiac troponin T

Higher absolute hs-cTn levels were associated with lower MoCA scores at baseline and at hospital discharge (adjusted beta − 2.619 (95% confidence interval − 4.045 - -1.193) and − 1.695 (95% confidence interval − 3.379 - -0.011) respectively; see Table 2). Higher absolute hs-cTn levels were also associated with cognitive impairment at baseline with an adjusted OR of 2.9 (95% CI 1.3–6.7, *p* = 0.012; also see Fig. 2) in logistic regression analysis.

**Fig. 3:**
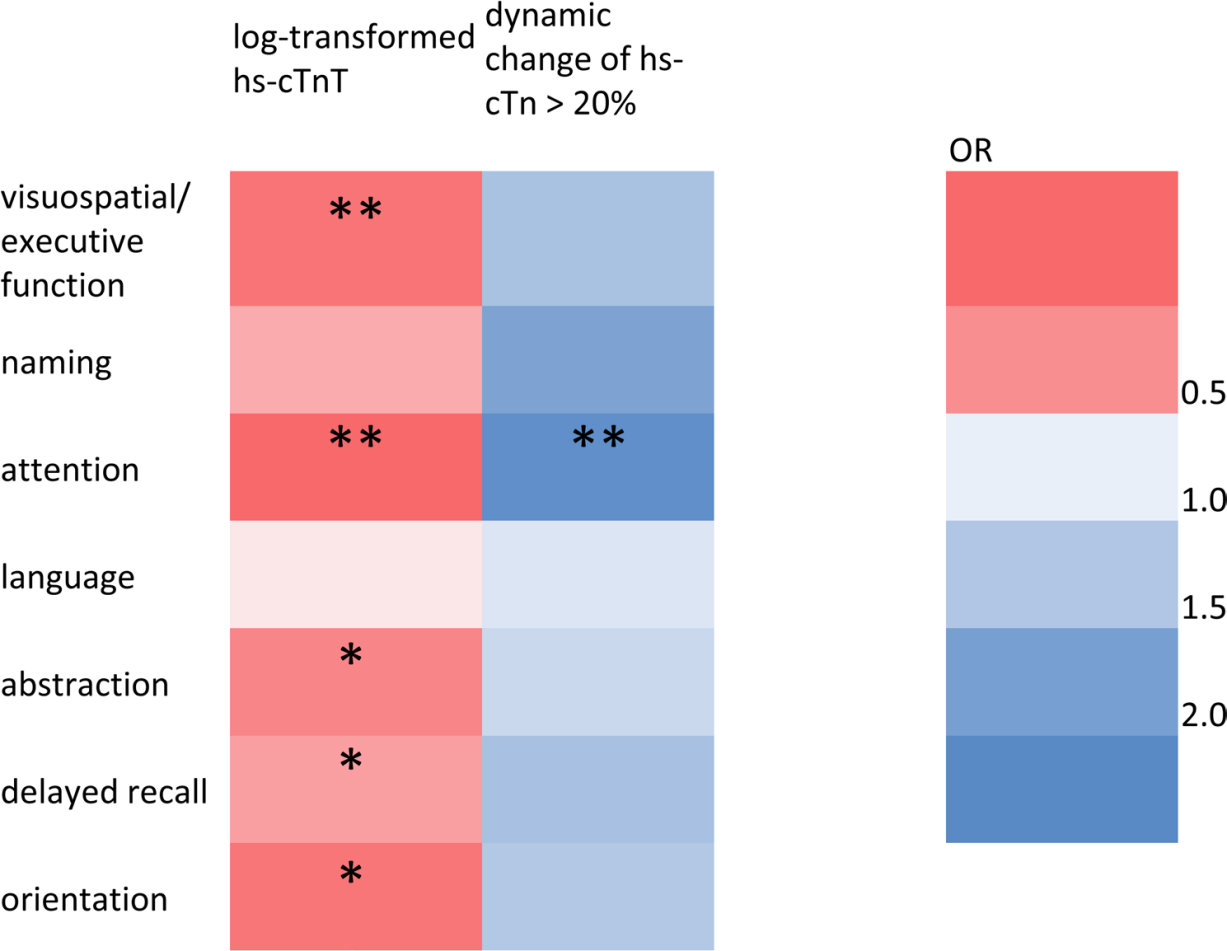
Associations between hs-cTn and constituent MoCA subscores at baseline. The figure includes odds ratios derived from ordinal regression adjusted for age, sex, diabetes, hypertension, coronary artery disease, heart failure, atrial fibrillation, baseline National Institutes of Health Stroke Scale, estimated glomerular filtration rate. Abbreviations: MoCA = Montreal Cognitive Assessment, hs-cTn = high-sensitivity cardiac troponin. *P < 0.05, **P < 0.01

In addition, we found an association between absolute initial hs-cTn levels and all MoCA subscores except language and naming (see Fig. 3 and supplemental table S2).

Acute myocardial injury (i.e. a dynamic change of hs-cTn values > 20%) was associated with higher MoCA scores at baseline but not at discharge (see Table 2) and with higher MoCA subscores in the domain “attention” (adjusted OR 2.2 (95% CI 1.3–3.7), *p* = 0.003; see Fig. 3 and supplemental table S2).

**Table 2:** Associations between hs-cTn and MoCA using linear regression analyses

	MoCA V1 (baseline)		MoCA V2 (discharge)	
	Unadjusted beta (95% CI)	Adjusted beta (95% CI)	Unadjusted beta (95% CI)	Adjusted beta (95% CI)
Severity of myocardial injury; Log(hs-cTnT) (*N* = 207)	***-2.776 (-4.246 - -1.307)***,*** p < 0.001***	***-2.638 (-4.074 - -1.201)***,*** p < 0.001***	***-1.995 (-3.691 - -0.299)***,*** p = 0.021***	***-1.695 (-3.379 - -0.011)***,*** p = 0.049***
Acute myocardial injury (dynamic change of hs-cTn > 20%)	***1.802 (0.392–3.211)***,*** p = 0.012***	***1.953 (0.580–3.326)***,*** p = 0.006***	0.347 (-1.236–1.929), *p* = 0.666	0.485 (-1.002–2.104), *p* = 0.485

Adjusted for age, sex, diabetes, hypertension, coronary artery disease, heart failure, atrial fibrillation, stroke severity (baseline National institutes of health stroke ScaleS), estimated glomerular filtration rate. Abbreviations: MoCA = Montreal cognitive Assessment, CI = confidence interval, hs-cTn = high-sensitivity cardiac troponin

### Hs-cTn and cognitive performance at follow-up

*N* = 151/254 (59%) patients had at least one available TICS score. The reasons for missing TICS scores are displayed in supplemental table S3. Patients with missing cognitive follow-up data were older, had a higher initial stroke severity and had worse cognitive performance at baseline (see Table 1). Cognitive performance did not change between three and twelve months after the index event: the median TICS score was 34 (IQR 32–36 points) and 35 (IQR 32–37 points) at three and twelve months, respectively. Neither absolute levels of hs-cTn, nor acute myocardial injury were associated with TICS scores at three and twelve months, respectively (see Table 3).

**Table 3:** Associations between hs-cTn and TICS using linear regression analyses

	TICS 3 months		TICS 12 months	
	Unadjusted beta (95% CI)	Adjusted beta (95% CI)	Unadjusted beta (95% CI)	Adjusted beta (95% CI)
Severity of myocardial injury; Log(hs-cTnT)	0.164 (-1.314–1.642), *p* = 0.827	-0.176 (-1.630–1.278), *p* = 0.811	-0.637 (-2.080–0.805), *p* = 0.383	-0.683 (-2.065–0.699), *p* = 0.328
Acute myocardial injury (dynamic change of hs-cTn > 20%)	0.208 (-1.012–1.427), *p* = 0.737	0.172 (-1.978–1.323), *p* = 0.767	-0.427 (-1.681–0.828), *p* = 0.502	-0.493 (-1.681–0.694), *p* = 0.412

Adjusted for age, sex, diabetes, hypertension, coronary artery disease, heart failure, atrial fibrillation, cognitive impairment at baseline (Montreal cognitive Assessment < 26 points), estimated glomerular filtration rate. Abbreviations: TICS = telephone interview for cognitive status, CI = confidence interval, hs-cTn = high-sensitivity cardiac troponin

Similarly, we found no association between hs-cTn and change of TICS over time in the longitudinal analyses (IPW GLM models, see Table 4).

**Table 4:** Associations between hs-cTn and TICS using generalized linear models with time-specific weights (IPW-GLM)

	unadjusted		Adjusted	
	Beta	95% CI	Beta	95% CI
Severity of myocardial injury; Log(hs-cTnT)	-0.170	-1.318–0.979 (*p* = 0.772)	-0.511	-1.656–0.632 (*p* = 0.380)
Acute myocardial injury (Dynamic change of hs-cTn > 20%)	-0.060	-1.210–1.090 (*p* = 0.919)	-0.040	-1.055–0.976 (*p* = 0.939)

Adjusted for age, sex, diabetes, hypertension, coronary artery disease, heart failure, atrial fibrillation, cognitive impairment at baseline (Montreal cognitive Assessment < 26 points), estimated glomerular filtration rate. Abbreviations: TICS = telephone interview for cognitive status, CI = confidence interval, hs-cTn = high-sensitivity cardiac troponin

## Discussion

In this pre-specified sub-analysis of the PRAISE study we investigated the association between both severity and acuteness of myocardial injury (hs-cTn elevation) and cognitive performance after ischemic stroke in patients with elevated hs-cTn levels both at time of stroke and during follow-up.

We found that more severe myocardial injury (characterized by higher absolute hs-cTn levels) was associated with cognitive impairment in cross-sectional analyses. This finding corroborates results of the PROSCIS study, which found that higher hs-cTnT levels were associated with poorer cognitive performance in patients with first-ever ischemic stroke [7]. Acute myocardial injury (i.e. elevated hs-cTn with a dynamic change >20%) was associated with a higher total MoCA score at baseline indicating better cognitive performance in these patients. However, we did not find an association with the MoCA score at discharge, which was obtained only a few days after study inclusion. To our knowledge, our study is the first to differentiate the impact of acute versus chronic myocardial injury on cognitive performance in stroke patients. Our study shows that cognitive impairment is associated with the severity of myocardial injury but not with acuteness of myocardial injury at the time of the index stroke. While acute myocardial injury is characterized by a rise-and-fall pattern of hs-cTn levels with a possible return to normal levels, chronic myocardial injury is characterized by stable elevation of hs-cTn levels over time. Therefore, patients with acute myocardial injury may have had highly elevated levels of hs-cTn for only a short period of time while patients with chronic myocardial injury likely had elevated levels of hs-cTn for a longer period of time. Thus, our results suggest that myocardial injury likely has to be present over a longer period of time to be associated with cognitive impairment. Moreover, patients with presence of acute myocardial injury had lower baseline levels of hs-cTn than patients with chronic myocardial injury. Therefore, the association between acute myocardial injury and better cognitive performance may also partly be explained by overall less severe myocardial injury (as indicated by lower absolute levels of hs-cTn) in this group.

Notably, cognitive impairment was very common in this selected cohort of stroke patients defined by presence of myocardial injury. Approximately 75% of the PRAISE study population had a MoCA score < 26 at baseline despite only mild to moderate stroke severity in most patients. This is considerably higher than the proportion of patients with cognitive impairment reported in other stroke cohorts where myocardial injury was no inclusion criterion [7, 20]. The association between severity of myocardial injury remained statistically significant with cognitive performance after adjustment for stroke severity. Thus, the high prevalence of cognitive impairment cannot be explained by severity of the index stroke but is suggestive of an independent association between (chronic) myocardial injury and cognitive impairment in patients with ischemic stroke. These associations with myocardial injury were strongest in the domains “attention” and “visuospatial/executive function”. These cognitive domains are typically affected in vascular cognitive impairment [21]. The finding is also supported by previous studies in the general population and in stroke patients that found a link between hs-cTnT and tests of attention and executive function [3, 4, 6].

We did not find an association between absolute levels of hs-cTn or dynamic change of hs-cTn and cognitive performance at follow-up measured by the TICS. This is in contrast to the results of the PROSCIS study, where higher levels of hs-cTn were associated with lower cognitive performance over three years [7]. However, our results regarding cognitive follow-up data should be interpreted with caution as there was a significant number of missing observations. The significant number of missing observations during follow-up taken together with the fact that patients with missing follow-up data tended to be older and more frequently had cognitive impairment at baseline may have biased the results of longitudinal analyses. A possible explanation for the high number of patients lost to follow-up in PRAISE is the frequent occurrence of adverse cardiovascular events (especially death) and poor functional outcome (see Table 1 and supplemental table S3).

The pathogenetic mechanisms underlying the association between hs-cTn and cognition are not yet fully understood. Potential underlying pathophysiological mechanisms include common cardiovascular risk factors leading to cerebral small vessel disease [6], systemic atherosclerosis [22, 23], microangiopathy [24], chronic cerebral hypoperfusion [23], cardioembolism [25], disruption of the blood-brain barrier [26] and systemic inflammation [27]. Since most of these proposed underlying mechanisms are chronic processes, it seems logical that the severity of myocardial injury rather than presence of acute myocardial injury in general is linked to poorer cognitive function.

To date, there is no specific treatment of myocardial injury in stroke patients. However, the primary results of the PRAISE study suggest that myocardial infarction is a common cause of hs-cTn elevation in stroke patients [11] and effective treatments for myocardial infarction are evident. Although PRAISE was a multicenter, prospective study with predefined endpoints applying validated measures, some limitations must be considered. First, all patients included in the study had elevated levels of hs-cTn. Thus, we had no control group of patients with normal hs-cTn levels. Patients with acute elevation of hs-cTn had lower initial absolute levels of hs-cTn than those without, which may have influenced our results and may explain the association between acute myocardial injury and higher initial MoCA scores. Second, study centers used different hs-cTn assays. To overcome variability in assays, we re-analyzed hs-cTn levels in a central core laboratory using the same hs-cTn assay. Third, pre-existing cognitive impairment was not an exclusion criterion in the PRAISE study. However, all patients included in PRAISE were able to give informed consent, excluding severe pre-stroke dementia. Fourth, it is likely that the results of cognitive tests were influenced by differences in the location of the stroke and stroke lesion characteristics (e.g. lesion size). However, as there was no central reading of cerebral imaging in PRAISE, we did not adjust our analyses for stroke localization or lesion size. Fifth, as in many prospective studies, there was missing data for cognitive follow-up assessments. Patients with missing cognitive follow-up data were older and more often had cognitive impairment at baseline. Thus, there was a bias toward better cognitive outcome among the participants who were followed-up. This may have attenuated the association between hs-cTn and cognitive performance during follow-up. Lastly, the follow-up period was 12 months after the index event and we cannot rule out effects on longer follow-up.

## Conclusions

In acute ischemic stroke patients, the severity but not the acuteness of myocardial injury is associated with cognitive impairment in cross-sectional analyses. Thus, assessing the severity of myocardial injury as indicated by absolute levels of hs-cTn seems to be most helpful in evaluating the risk of cognitive impairment in acute ischemic stroke patients. The association with myocardial injury seems to be more pronounced in executive function and attention, which are typically affected in patients with vascular cognitive impairment. This suggests that the severity of myocardial injury is associated with the degree of chronic systemic vascular damage (including to the brain), which facilitates cognitive impairment. Future studies may utilize novel neuroimaging markers (e.g. perfusion imaging or diffusion tensor imaging) to explore the significance of different pathophysiological mechanisms underlying the link between hs-cTn and cognitive impairment. Additionally, future studies may investigate the potential value of hs-cTn in monitoring therapeutic effects in prevention and treatment of vascular cognitive impairment.

## Supplementary Information

Below is the link to the electronic supplementary material.

Supplementary Material 1

## Data Availability

The datasets analyzed during the current study are not publicly available due to data protection regulations but are available from the corresponding author on reasonable request.

## References

[1] Pendlebury, S. T., Rothwell, P. M., & Oxford Vascular, S. (2019). Incidence and prevalence of dementia associated with transient ischaemic attack and stroke: Analysis of the population-based Oxford vascular study. *Lancet Neurology*, *18*, 248–25820190212. 10.1016/S1474-4422(18)30442-3 30784556 10.1016/S1474-4422(18)30442-3 PMC6390174

[2] Rusanen, M., Kivipelto, M., Levalahti, E., et al. (2014). Heart diseases and long-term risk of dementia and alzheimer’s disease: A population-based CAIDE study. *Journal of Alzheimer’S Disease*, *42*, 183–191. 10.3233/JAD-132363 24825565 10.3233/JAD-132363

[3] von Rennenberg, R., Liman, T., Nolte, C. H., et al. (2023). High-Sensitivity cardiac troponin T and cognitive decline in older adults: Results of the Berlin aging study II. *Gerontology*, *69*(20220505), 140–148. 10.1159/000523845 35512662 10.1159/000523845

[4] Schneider, A. L., Rawlings, A. M., Sharrett, A. R., et al. (2014). High-sensitivity cardiac troponin T and cognitive function and dementia risk: The atherosclerosis risk in communities study. *European Heart Journal*, *35*, 1817–1824. 10.1093/eurheartj/ehu124 24685712 10.1093/eurheartj/ehu124 PMC4097965

[5] Scheitz, J. F., Nolte, C. H., Doehner, W., et al. (2018). Stroke-heart syndrome: Clinical presentation and underlying mechanisms. *Lancet Neurology*, *17*, 1109–112020181026. 10.1016/S1474-4422(18)30336-3 30509695 10.1016/S1474-4422(18)30336-3

[6] von Rennenberg, R., Nolte, C. H., Liman, T. G., et al. (2024). High-sensitivity cardiac troponin T and cognitive function over 12 months after stroke-results of the DEMDAS study. *J Am Heart Assoc*, *13*, e033439. 10.1161/JAHA.123.033439 38456438 10.1161/JAHA.123.033439 PMC11010029

[7] Broersen, L. H. A., Siegerink, B., Sperber, P. S., et al. (2020). High-sensitivity cardiac troponin T and cognitive function in patients with ischemic stroke. *Stroke*, *51*, 1604–160720200413. 10.1161/STROKEAHA.119.028410 32279621 10.1161/STROKEAHA.119.028410

[8] Stengl, H., Ganeshan, R., Hellwig, S., et al. (2022). Frequency, associated variables, and outcomes of acute myocardial injury according to the fourth universal definition of myocardial infarction in patients with acute ischemic stroke. *Eur Stroke J*, *7*(20221013), 413–420. 10.1177/23969873221120159 36478763 10.1177/23969873221120159 PMC9720848

[9] Thygesen, K., Alpert, J. S., Jaffe, A. S., et al. (2018). Fourth universal definition of myocardial infarction (2018). *Journal of the American College of Cardiology*, *72*, 2231–2264. 10.1016/j.jacc.2018.08.1038 30153967 10.1016/j.jacc.2018.08.1038

[10] Scheitz, J. F., Mochmann, H. C., Erdur, H., et al. (2014). Prognostic relevance of cardiac troponin T levels and their dynamic changes measured with a high-sensitivity assay in acute ischaemic stroke: Analyses from the TRELAS cohort. *International Journal of Cardiology*, *177*, 886–893. 10.1016/j.ijcard.2014.10.036 25453407 10.1016/j.ijcard.2014.10.036

[11] Nolte, C. H., von Rennenberg, R., Litmeier, S., et al. (2024). Type 1 myocardial infarction in patients with acute ischemic stroke. *JAMA Neurol*, *81*, 703–711. 10.1001/jamaneurol.2024.1552 38829625 10.1001/jamaneurol.2024.1552 PMC11148785

[12] Nolte, C. H., von Rennenberg, R., Litmeier, S., et al. (2020). PRediction of acute coronary syndrome in acute ischemic stroke (PRAISE) - protocol of a prospective, multicenter trial with central reading and predefined endpoints. *Bmc Neurology*, *20*, 318. 10.1186/s12883-020-01903-0 32854663 10.1186/s12883-020-01903-0 PMC7450553

[13] Adams, H. P. Jr., Davis, P. H., Leira, E. C., et al. (1999). Baseline NIH stroke scale score strongly predicts outcome after stroke: A report of the trial of org 10172 in acute stroke treatment (TOAST). *Neurology*, *53*, 126–131. 10.1212/wnl.53.1.126 10408548 10.1212/wnl.53.1.126

[14] Collet, J. P., Thiele, H., Barbato, E., et al. (2021). 2020 ESC guidelines for the management of acute coronary syndromes in patients presenting without persistent ST-segment elevation. *European Heart Journal*, *42*, 1289–1367. 10.1093/eurheartj/ehaa575 32860058 10.1093/eurheartj/ehaa575

[15] Nasreddine, Z. S., Phillips, N. A., Bedirian, V., et al. (2005). The montreal cognitive assessment, moca: A brief screening tool for mild cognitive impairment. *Journal of the American Geriatrics Society*, *53*, 695–699. 10.1111/j.1532-5415.2005.53221.x 15817019 10.1111/j.1532-5415.2005.53221.x

[16] Rost, N. S., Brodtmann, A., Pase, M. P., et al. (2022). Post-stroke cognitive impairment and dementia. *Circ Res*, *130*(20220414), 1252–1271. 10.1161/CIRCRESAHA.122.319951 35420911 10.1161/CIRCRESAHA.122.319951

[17] Pendlebury, S. T., Welch, S. J., Cuthbertson, F. C., et al. (2013). Telephone assessment of cognition after transient ischemic attack and stroke: Modified telephone interview of cognitive status and telephone Montreal cognitive assessment versus face-to-face Montreal cognitive assessment and neuropsychological battery. *Stroke*, *44*, 227–22920121108. 10.1161/STROKEAHA.112.673384 23138443 10.1161/STROKEAHA.112.673384 PMC5593099

[18] Zietemann, V., Kopczak, A., Muller, C., et al. (2017). Validation of the telephone interview of cognitive status and telephone Montreal cognitive assessment against detailed cognitive testing and clinical diagnosis of mild cognitive impairment after stroke. *Stroke*, *48*, 2952–2957. 10.1161/STROKEAHA.117.017519 29042492 10.1161/STROKEAHA.117.017519

[19] Daza, E. J., Hudgens, M. G., & Herring, A. H. (2017). Estimating inverse-probability weights for longitudinal data with dropout or truncation: The Xtrccipw command. *Stata J*, *17*, 253–278. 29755297 PMC5947963

[20] Georgakis, M. K., Fang, R., During, M., et al. (2023). Cerebral small vessel disease burden and cognitive and functional outcomes after stroke: A multicenter prospective cohort study. *Alzheimers Dement*, *19*, 1152–1163. 10.1002/alz.12744 35876563 10.1002/alz.12744

[21] Salmon, D. P. (2012). Neuropsychological features of mild cognitive impairment and preclinical alzheimer’s disease. *Current Topics in Behavioral Neurosciences*, *10*, 187–212. 10.1007/7854_2011_171 22042707 10.1007/7854_2011_171

[22] Saunders, J. T., Nambi, V., de Lemos, J. A., et al. (2011). Cardiac troponin T measured by a highly sensitive assay predicts coronary heart disease, heart failure, and mortality in the atherosclerosis risk in communities study. *Circulation*, *123*, 1367–1376. 10.1161/CIRCULATIONAHA.110.005264 21422391 10.1161/CIRCULATIONAHA.110.005264 PMC3072024

[23] Kim, B. J., Lee, S. H., Kim, C. K., et al. (2011). Advanced coronary artery calcification and cerebral small vessel diseases in the healthy elderly. *Circulation Journal*, *75*(20101210), 451–456. 10.1253/circj.cj-10-0762 21157110 10.1253/circj.cj-10-0762

[24] Zhang, X., & Le, W. (2010). Pathological role of hypoxia in Alzheimer’s disease. *Experimental Neurology*, *223*, 299–303. 10.1016/j.expneurol.2009.07.033 19679125 10.1016/j.expneurol.2009.07.033

[25] Psaty, B. M., Manolio, T. A., Kuller, L. H., et al. (1997). Incidence of and risk factors for atrial fibrillation in older adults. *Circulation*, *96*, 2455–2461. 10.1161/01.cir.96.7.2455 9337224 10.1161/01.cir.96.7.2455

[26] Hachinski, V., Einhaupl, K., Ganten, D., et al. (2019). Preventing dementia by preventing stroke: The Berlin manifesto. *Alzheimers Dement*, *15*, 961–984. 10.1016/j.jalz.2019.06.001 31327392 10.1016/j.jalz.2019.06.001 PMC7001744

[27] Zonneveld, M. H., Trompet, S., Jukema, J. W., et al. (2023). Exploring the possible causal effects of cardiac blood biomarkers in dementia and cognitive performance: A Mendelian randomization study. *Geroscience*, *45*, 3165–317420230513. 10.1007/s11357-023-00814-5 37178386 10.1007/s11357-023-00814-5 PMC10643774

