# Supplementary material for: Cognitive performance in patients with ischemic stroke and additional myocardial injury – results from the multicenter prospective observational PRAISE study
Source: Neurol Res Pract. 2025 Nov 4;7(1):84. doi: 10.1186/s42466-025-00446-4 (PMC12587579; doi:10.1186/s42466-025-00446-4)
Supplement: Supplementary file 1 — Supplementary Material 1 [file 42466_2025_446_MOESM1_ESM.docx]

**Association between acute and chronic myocardial injury and cognitive performance in acute ischemic stroke – results from the multicenter prospective observational PRAISE study**

**Supplement material**

| **Domain** | **Items/maximum scores** | **Maximum domain subscores** |
| --- | --- | --- |
| Visuospatial/executive function | Trail making test/1 | 5 |
|  | Copy cube/1 |  |
|  | Clock drawing/3 |  |
| Naming | Animal naming/3 | 3 |
| Attention | Serial substractions/3 | 5 |
|  | Test for letter “A”/1 |  |
|  | Digit span/2 |  |
| Language | Verbal fluency/1 | 3 |
|  | Repetition of two complex sentences/2 |  |
| Abstraction | Similarities/2 | 2 |
| Delayed recall | Recall a list of five words/5 | 5 |
| Orientation | Orientation to year, month, day, weekday, place, city | 6 |

**Supplemental table S1. Overview of the constituent MoCA subscores**

|  | Executive function | Naming | Attention | Language | Abstraction | Delayed recall | Orientation |
| --- | --- | --- | --- | --- | --- | --- | --- |
| Log(hs-cTnT) unadjusted | ***0.475 (0.279-0.808), p=0.006*** | 0.566 (0.270-1.186), p=0.132 | ***0.442 (0.257-0.761), p=0.003*** | 0.755 (0.425-1.339), p=0.336 | ***0.510 (0.274-0.949), p=0.033*** | ***0.536 (0.312-0.923), p=0.025*** | ***0.464 (0.250-0.860), p=0.015*** |
| Log (hs-cTnT) adjusted | ***0.424 (0.238-0.753), p=0.003*** | 0.589 (0.259-1.338), p=0.206 | ***0.393 (0.219-0.707), p=0.002*** | 0.774 (0.424-1.412), p=0.404 | ***0.467 (0.242-0.902), p=0.023*** | ***0.553 (0.315-0.969), p=0.038*** | ***0.424 (0.214-0.840), p=0.014*** |
| Dynamic change of hs-cTn > 20% unadjusted | 1.238 (0.773-1.984), p=0.375 | 1.876 (0.948-3.710), p=0.071 | ***2.099 (1.280-3.442), p=0.003*** | 1.046 (0.641-1.709), p=0.856 | 1.387 (0.811-2.372), p=0.232 | ***1.624 (1.005-2.622), p=0.048*** | 1.176 (0.655-2.110), p=0.588 |
| Dynamic change of hs-cTn > 20% adjusted | 1.564 (0.943-2.594), p=0.083 | 2.096 (0.999-4.398), p=0.050 | ***2.274 (1.351-3.827), p=0.002*** | 1.169 (0.699-1.955), p=0.553 | 1.364 (0.779-2.390), p=0.277 | 1.622 (0.978-2.689), p=0.061 | 1.460 (0.767-2.781), p=0.249 |

**Supplemental Table S2. Assocation between hs-cTn and the constituent MoCA subscores at baseline using ordinal regression models.** Adjusted for age, sex, diabetes, hypertension, coronary artery disease, heart failure, atrial fibrillation, baseline National Institutes of Health Stroke Scale, estimated glomerular filtration rate. Abbreviations: MoCA = Montreal Cognitive Assessment, hs-cTnT = high-sensitivity cardiac troponin T

|  | **TICS 3 months** | **TICS 12 months** |
| --- | --- | --- |
| Death | 32 | 42 |
| Patient lost to follow-up | 28 | 29 |
| Patient unable to undergo cognitive interview due to health-related reasons | 34 | 22 |
| Interview declined | 17 | 31 |
| Total number of missing observations | 111 | 131 |

**Supplemental Table S3. Number of missing TICS scores after 3 and 12 months by reason of missingness**

Abbreviations: TICS = telephone interview for cognitive status
